# Insulin secretion in patients with latent autoimmune diabetes (LADA): half way between type 1 and type 2 diabetes: action LADA 9

**DOI:** 10.1186/1472-6823-15-1

**Published:** 2015-01-09

**Authors:** Marta Hernandez, Angels Mollo, Josep Ramon Marsal, Aureli Esquerda, Ismael Capel, Manel Puig-Domingo, Paolo Pozzilli, Alberto de Leiva, Didac Mauricio, on behalf of the Action LADA consortium

**Affiliations:** Department of Endocrinology and Nutrition, Hospital Universitari Arnau de Vilanova, Lleida, Spain; Institut de Recerca Biomedica de Lleida, Universitat de Lleida, Lleida, Spain; Centre d’Atenció Primària de Cervera, Institut Català de la Salut, Lleida, Spain; Unitat de Suport a la Recerca de Lleida, Institut Universitari d’Investigació en Atenció Primària Jordi Gol, Lleida, Spain; Unidad de Epidemiologia, Servicio de Cardiología, Hospital Universitari Vall Hebrón, Barcelona, Spain; Universitat Autònoma de Barcelona, Bellaterra, Barcelona, Spain; Clinical Laboratory, Hospital Universitari Arnau de Vilanova, Lleida, Spain; Department of Endocrinology and Nutrition, Hospital de Sant Pau - IIB-Sant Pau, Barcelona, Spain; CIBER de Bioingeniería, Biomateriales y Nanomedicina, Barcelona, Spain; Department of Endocrinology and Nutrition, Hospital Univesitari Germans Trias i Pujol, Carretera Canyet, S/N, 08916 Badalona, Spain; Department of Endocrinology and Diabetes, University Campus Bio-Medico, Rome, Italy; Centre of Diabetes, Barts and the London School of Medicine, Queen Mary University of London, London, UK

**Keywords:** Latent autoimmune diabetes in adults, Type 1 diabetes mellitus, Type 2 diabetes mellitus, Insulin secretion, C-peptide

## Abstract

**Background:**

The study of endogenous insulin secretion may provide relevant insight into the comparison of the natural history of adult onset latent autoimmune diabetes (LADA) with types 1 and 2 diabetes mellitus. The aim of this study was to compare the results of the C-peptide response to mixed-meal stimulation in LADA patients with different disease durations and subjects with type 2 and adult-onset type 1 diabetes.

**Methods:**

Stimulated C-peptide secretion was assessed using the mixed-meal tolerance test in patients with LADA (n = 32), type 1 diabetes mellitus (n = 33) and type 2 diabetes mellitus (n = 30). All patients were 30 to 70 years old at disease onset. The duration of diabetes in all groups ranged from 6 months to 10 years. The recruitment strategy was predefined to include at least 10 subjects in the following 3 disease onset categories for each group: 6 to 18 months, 19 months to 5 years and 5 to 10 years.

**Results:**

At all time-points of the mixed-meal tolerance test, patients with LADA had a lower stimulated C-peptide response than the type 2 diabetes group and a higher response than the type 1 diabetes group. The same results were found when the peak or area under the C-peptide curve was measured. When the results were stratified by time since disease onset, a similar pattern of residual insulin secretory capacity was observed.

**Conclusions:**

The present study shows that the magnitude of stimulated insulin secretion in LADA is intermediate between that of type 1 and type 2 diabetes mellitus.

**Electronic supplementary material:**

The online version of this article (doi:10.1186/1472-6823-15-1) contains supplementary material, which is available to authorized users.

## Background

The increased frequency of insulin treatment in adult patients with latent autoimmune diabetes (LADA) is a result of an accelerated pancreatic beta-cell loss due to autoimmune damage [1]. The measurement of the endogenous insulin secretion may provide relevant insight into the comparison of the natural history of LADA with the natural histories of types 1 and 2 diabetes; however, limited data are available regarding fasting or stimulated C-peptide secretion in LADA [2–8]. Stimulated C-peptide is currently accepted as the reference outcome for assessing beta-cell secretory response in trials studying the prevention of beta-cell loss in established autoimmune diabetes [9]. In addition, no studies have assessed the insulin secretory response using the standardised mixed-meal tolerance test (MMTT) in patients with LADA. Therefore, the aim of our study was to assess the stimulated C-peptide response in LADA patients with different disease durations and to compare the results to those of subjects with type 2 and adult-onset type 1 diabetes.

## Methods

### Participants and clinical data

In this cross-sectional study, we enrolled 32 subjects with LADA, 33 subjects with type 1 and 30 subjects with type 2 diabetes mellitus. A total of 11 study subjects, 4 with LADA and 7 with type 1 diabetes, were recruited in Hospital de Sant Pau. All other subjects were included at Hospital Arnau de Vilanova. All study subjects were 30 – 70 years old at the time of diagnosis. Type 1 and type 2 diabetes mellitus were diagnosed according to the standard criteria as described previously [8], and LADA was defined as patients aged 30 – 70 years at the diagnosis of diabetes who did not require insulin for at least 6 months after diagnosis, with glutamic acid decarboxylase (GAD) autoantibody (GADAb) or tyrosine phosphatase autoantibody (IA-2Ab) positivity. The study was initially part of the Action LADA project that was extended locally for an additional period of 3 years [10]. The study protocol was designed to recruit 10 subjects in the following disease duration categories for each group: 6 – 18 months, 19 months – 5 years, and 5 – 10 years from diagnosis. This number of subjects was established based on previous studies that yielded clear differences when this sample size was used to compare LADA and type 2 diabetes [1, 2]. Thus, at least 30 patients of each diabetes type were included. None of the subjects had impaired kidney function, liver disease or any other condition known to interfere with the C-peptide response. Patients were included consecutively after they signed a written informed consent document. The study protocol was approved by the local Ethics Committee of the two centers where patients were recruited, i.e. Hospital de Sant Pau (Barcelona) and Hospital Arnau de Vilanova (Lleida).

For each subject, age, sex, weight, height, body mass index, and disease duration were obtained as recently described [8]. The time to start of insulin was calculated as the time between the date of diagnosis and the date of the first insulin treatment. In addition, glucose and HbA1c levels were tested using standard laboratory assays. A detailed description of the methods was recently published [8].

### Antibody measurements

Antibodies to islet cell antigens (GADA65 and IA-2) were measured with two commercially available ELISA kits: GAD65 Autoantibody ELISA and IA-2 Autoantibody ELISA (both from DRG Diagnostic, Marburg, Germany), as previously described [11, 12]. Optimal cut-off values for positivity were set at 5 and 15 U/mL for GADAb and IA-2Ab, respectively. Both assays showed good performance when tested in the Diabetes Antibody Standardization Program Workshops. In the Diabetes Antibody Standardization Program Workshop 2007, sensitivities and specificities were 94% and 97% for GADAb, respectively, and 66% and 95% for IA-2Ab; in the Diabetes Antibody Standardization Program Workshop 2009, the figures were 82% and 95% for GADAb, and 60% and 100% for IA-2Ab, respectively.

### Mixed meal tolerance test

The test was performed following the same procedures as those described in the European C-Peptide Trial in which our group participated [13]. Briefly, after an overnight fasting period, all study subjects were scheduled to start the test before 10 a.m. the next morning. Subjects were asked to withhold any short-acting insulin for at least 6 hours before the test. The standard meal consisted of 6 ml/kg of a Boost HP drink (Nestle Nutrition, Vevey, Switzerland); the subjects were given up to 360 ml for up to 5 minutes. C-peptide samples were collected at times -5 and 0 minutes before (the mean of these two values was taken as the fasting concentration), and 10, 20, 30, 60, 90 and 120 minutes after the final intake of the mixed-meal.

C-peptide was measured by a chemiluminescence assay (Immulite 2000, Siemens Medical Solutions Diagnostics, LA, USA), with reference values of 0.30 – 2.4 nmol/l (intra and interassay coefficients of variation were less than 5%). The lower detection limit was 0.03 nmol/l. Any value below the lower detection limit was arbitrarily assigned a concentration of 0.029 nmol/l.

### Statistical analysis

Continuous variables are expressed as the mean (± SD) or the median (interquartile range ([IQR]) where appropriate. Categorical variables are described using the absolute and relative frequencies. Variables with a skewed distribution were log-transformed before analysis. Comparisons between groups were performed using the non-parametric Mann–Whitney *U* test for numerical variables and the *χ*^2^ test for percentages. The area under the curve of C-peptide (AUCCP) was computed from all timed collections using the trapezoidal rule [9]. The analyses of the AUCCP and peak C-peptide were adjusted for sex, body mass index, age, duration of diabetes, fasting glucose, HbA1c and basal C-peptide. The results were considered to be statistically significant if the two-sided p-value was less than 0.05. All analyses were performed with SPSS for Windows version 15.0 (Chicago, USA).

## Results

The clinical characteristics of the study groups are shown in Table 1. There were no differences in terms of age, sex distribution and disease duration among the 3 study groups. As already found in previous studies in our setting [8], type 2 diabetic subjects demonstrated better glycemic control than patients with LADA and type 1 diabetes. In addition, the frequency of insulin treatment was higher in LADA subjects than in type 2 diabetic subjects.

**Table 1:** **Clinical characteristics and C-peptide stimulated secretion of the 3 study groups**

	Type 1 diabetes	Type 2 diabetes	LADA	p ^a^	p ^b^
Women, n (%)	17 (51.5%)	9 (30%)	11 (34.4%)	0.166	0.715
Age, years	46.9 (10.6)	53.7 (12.9)	49.6 (10.6)	0.170	0.260
Disease duration, months^c^	31 [12–56]	31 (10 – 83)	38.5 [13.5 – 59]	0.618	0.897
BMI, kg/m^2^	24.7 (4.2)	30.1 (5.5)	27.9 (4.8)	0.004	0.110
Fasting glucose, mmol/l	9.0 (3.49)	7.44 (1.92)	8.84 (3.45)	0.929	0.147
HbA1c, %	7.5 (1.4)	6.9 (1.0)	8.3 (2.4)	0.265	0.012
HbA1c, mmol/mol	58.4 (15.5)	52.2 (11.4)	67.6 (26.7)		
Insulin treatment, n (%)	33 (100%)	8 (26.7%)	20 (62.5%)	<0.001	0.005
Sulphonylurea treatment, n (%)	-	8 (20%)	5 (15.6%)	-	0.652
Categories, n				0.867	0.765
6 – 18 m	10	10	10		
19 m – 5 years	12	10	10		
5 – 10 years	11	10	12		
AUC C-peptide, nmol/l^d^	41.3 (46.4)	275.5 (121.2)	140.8 (102.2)	<0.001	<0.001
	19.2 (16.8 – 54.9)	245.1 (182.6 – 370.8)	112.1 (54.6 – 231.2)		
Peak C-peptide, nmol/l^c,d^	0.45 (0.50)	3.01 (1.29)	1.50 (1.05)	<0.001	<0.001
	0.16 (0.16 – 0.60)	2.54 (2.18 – 3.67)	1.44 (0.68 – 2.32)		

^a^p value for comparisons between type 1 diabetes and LADA; ^b^p value for comparison between type 2 diabetes and LADA; ^c^distributed variables; ^d^peak and AUC C-peptide results are given as mean (SD) and also as median (IQR).

Concerning treatment of hyperglycemia, insulin treatment was more frequent in LADA than in type 2 diabetes (62.5% vs. 26.7; p = 0.005) (Table 1). The distribution of the number of insulin-treated type 2 diabetic subjects according to disease duration was 2, 3 and 3 in categories 6 –18 months, 19 months – 5 years, and 5 – 10 years, respectively. The corresponding distribution of insulin treatment in patients with LADA was 5, 6 and 9 in disease duration categories 6 –18 months, 19 months – 5 years, and 5 – 10 years, respectively. The frequency of sulphonylurea drug use was similar in patients with type 2 diabetes and LADA (not significantly different). Six type 2 diabetic subjects were under sulphonylureas, all with a disease duration longer than 5 years. Five patients with LADA were under sulphonylurea treatment (3 in the disease duration category 19 months – 5 years, and 2 in the duration category 5 – 10 years). Only one patient with LADA was treated with sitagliptin, without any other study subjects treated with a dipeptidylpeptidase – 4 inhibitor.

**Figure 1:**
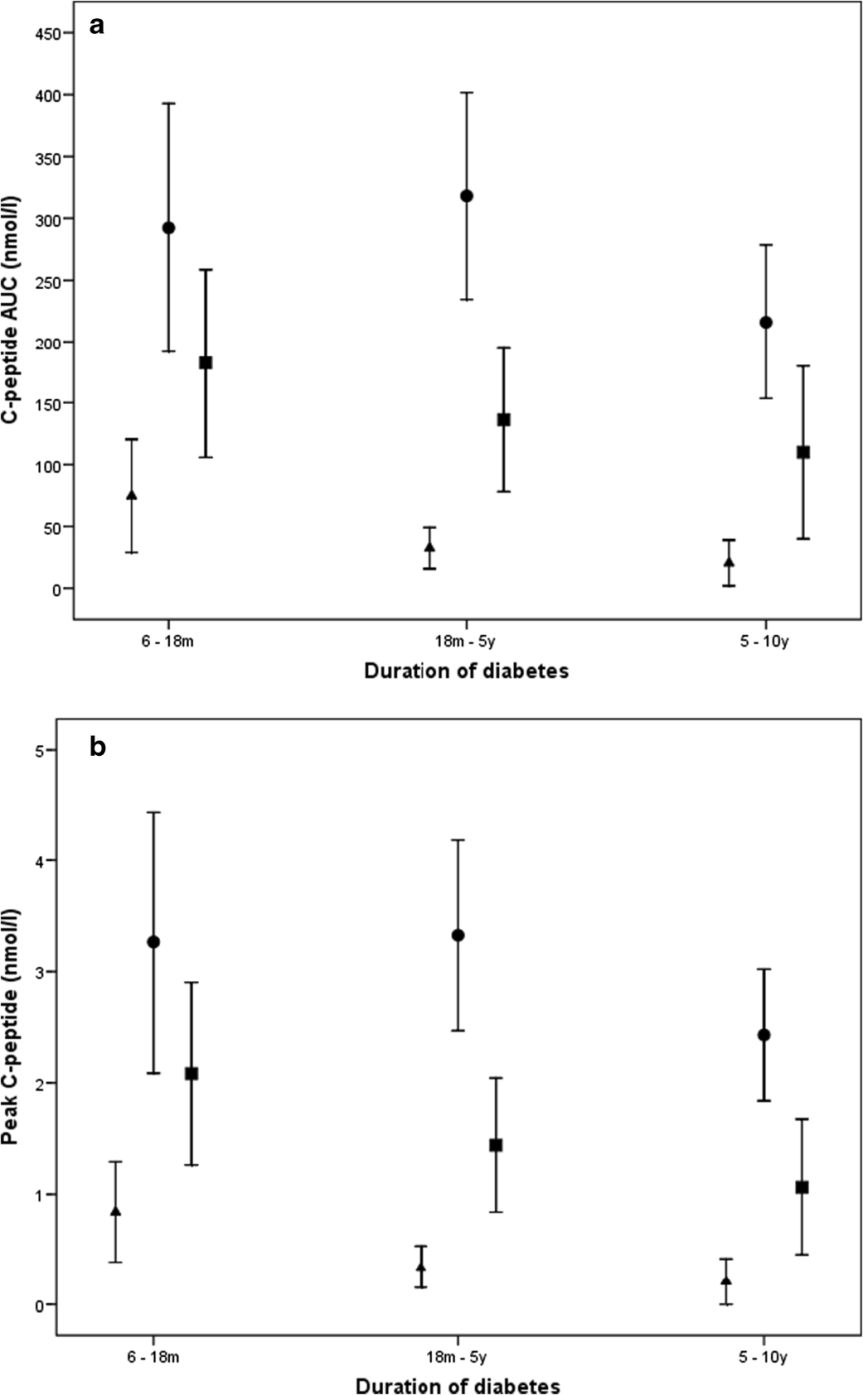
**The results of the mixed-meal tolerance test (panel a: area under the curve; panel b: peak C-peptide) of the different study groups (33 with type 2, 32 with LADA and 30 with type 2 diabetes), stratified according to the duration of diabetes mellitus.** Footnote: Triangles, circles and squares denote mean values with 95% CI of C-peptide in type 1 diabetes, type 2 diabetes and LADA, respectively. The differences between LADA and type 1 and type 2 diabetes are statistically significant except for the comparison of the peak and AUCCP of LADA and type 2 diabetes in subjects with a shorter diabetes duration (6 to 18 months). The number of patients treated with insulin was 8 in the group of type 2 diabetes and 20 in the group of LADA. For further details, see Table 1, and Additional files 1 and 2.

Subjects with LADA showed a capacity of stimulated C-peptide secretion that is intermediate between types 1 and 2 diabetic patients, both in terms of the peak secretion and the AUCCP (Table 1). In LADA, these measures of insulin secretion were significantly lower than in type 2 diabetic subjects and significantly higher than in type 1 subjects. These differences remained unchanged after adjusting for other confounding variables as age, sex, body mass index, HbA1c and glucose. This lower residual insulin secretion capacity was also confirmed when subjects were stratified by disease duration (Figure 1, and Additional files 1 and 2). Detailed results for AUCCP and peak C-peptide secretion according to disease duration are shown in Additional files 1 and 2. Most measures of C-peptide secretion were significantly different between patients with LADA and those with type 1 or type 2 diabetes; the only exception was the comparison between the sub-analysis of the peak C-peptide and AUCCP of LADA and type 2 diabetes in subjects with shorter diabetes duration (6 to 18 months).

Concerning the stimulated C-peptide in insulin-treated patients, those with LADA showed a lower response that was lower than that of type 2 diabetic subjects. Patients with LADA showed an AUCCP (102.4 ± 112.3 nmol/l) and peak C-peptide (1.25 ± 1.08 nmol/l) that were below those of type 2 diabetes patients (AUCPP: 200.1 ± 91.7 nmol/l; peak C-peptide: 2.23 ± 0.85 nmol/l) (p = 0.043 and p = 0.038, respectively). This finding is specially relevant considering that the disease duration of this 2 sub-groups of insulin-treated patients is not different (type 2 diabetes: 45.6 ± 42.8 months; LADA: 46 ± 32.2 months). Concerning non-insulin treated patients, those with LADA also had a lower C-peptide response, with an AUCCP (174.7 ± 74.8 nmol/l) and peak C-peptide (1.92 ± 0.87 nmol/l) in comparison with type 2 diabetic patients (AUCPP: 302.9 ± 120.5 nmol/l; peak C-peptide: 3.29 ± 1.32 nmol/l) (p = 0.002 and p = 0.001, respectively).

## Discussion

The current findings clearly show that subjects with LADA, as defined in the Action LADA, have a different capacity of meal-stimulated insulin secretion at different disease stages. This is the first study to use the standardised MMTT to assess the residual stimulated insulin secretion in LADA patients based on different periods of disease duration.

The current findings are consistent with some previous studies in the literature. The fasting C-peptide results are similar to the values found in previous studies [3, 4, 6, 8]. The current differences in fasting C-peptide confirm our previous findings in a larger population sample [8]. Also, the results show a consistent lower stimulated C-peptide secretion in those patients with LADA with or without insulin-treatment with respect to the respective counterparts with type 2 diabetes. Hosszúfalusi et al. also described the same initial pattern of differences with type 1 and type 2 diabetes; however, C-peptide secretion in patients with longer-duration LADA overlapped with the values for type 2 diabetic patients. In addition, over 6 years of follow-up, two prospective cohort studies with subjects of Asian origin consistently showed a more rapid loss of insulin secretion in LADA, measured as fasting C-peptide, than in type 2 diabetes [4, 6].

However, the most relevant information concerning residual insulin secretion in patients with LADA comes from Swedish studies. A study by Carlsson et al. demonstrated a decreased glucagon- and arginine-stimulated C-peptide secretion in a small group of LADA patients when compared to antibody-negative type 2 diabetic subjects [2]. Previously, the most relevant follow-up data of stimulated insulin secretion were described in sub-studies of a Swedish cohort of type 2 diabetic subjects [1]. In this group glucose- and glucagon-stimulated insulin secretion were clearly different in autoimmune diabetes when compared to antibody negative type 2 diabetic patients up to 3 years after diagnosis. A comparison with type 1 diabetic subjects demonstrated improved insulin secretion in LADA that subsequently disappeared after 1 year of follow-up. In addition, data from a recent Swedish intervention study showed that during 36 months of follow-up, the glucagon stimulated C-peptide response decreased in patients with conventionally treated LADA [7].

The main limitation of this study is its cross-sectional design. Ideally, the characterisation of the natural history of insulin secretion in LADA subjects would be analysed in prospective follow-up studies. Based on the results of the current study, a prospective characterisation of the time-course of the loss of the insulin secretory capacity and the comparison with other diabetes types would require a multi-centre study with long-term follow-up. Another limitation is the number of subjects studied; however, this is the first study to use the reference MMTT method to compare the stimulated C-peptide response in LADA to the responses in types 1 and 2 diabetes on different stages of disease duration.

## Conclusions

The present study shows that the stimulated insulin secretion capacity in LADA is intermediate between that of types 1 and 2 diabetes. This finding provides new data and further contributes to the characterisation of the natural history of LADA.

In addition, the study results may be helpful for future intervention trials aimed at preserving the residual insulin secretory capacity in subjects with autoimmune diabetes. Potential future prevention trials should be designed accordingly.

## Authors’ information

Members of the Action LADA Group:

Professor Richard David Leslie, Dr Mohammed I Hawa, Blizard Institute, Queen Mary University of London, London, UK.

Professor Paolo Pozzilli MD, University Campus Bio-Medico, Rome.

Professor Rhys Williams MD, Dr Sinead Brophy PhD Swansea University, Swansea.

Professor Henning Beck-Nielsen MD and Dr Knud Yderstraede MD, University Hospital of Odense, Odense.

Dr Steven Hunter, MD and Professor David Hadden MD, Royal Victoria Hospital, Belfast.

Professor Raffaella Buzzetti MD, University La Sapienza, University of Rome.

Professor Werner Scherbaum MD and Professor Hubert Kolb, PhD, University of Düsseldorf, Düsseldorf.

Dr Nanette C. Schloot, MD German Diabetes Centre, University of Düsseldorf, Düsseldorf and Clinic for Metabolic Diseases at University Hospital Düsseldorf. N.Schloot is on leave of absence from the German Diabetes Center and is currently employed by Lilly Deutschland, Bad Homburg, Germany.

Dr Jochen Seissler, MD, Ludwig-Maximilians-University, Munich.

Professor Guntram Schernthaner MD, Rudolfstiftung Hospital, Vienna.

Professor Jaakko Tuomilehto MD and Dr Cinzia Sarti MD, National Institute for Health and Welfare, Helsinki,

Professor Alberto De Leiva PhD, and Dr Eulalia Brugues MSc Universitat Autonoma de Barcelona, Barcelona.

Dr Didac Mauricio MD, University Hospital Germans Trias Pujol, Badalona, Spain.

Professor Charles Thivolet, MD, Hopital Edouard Herriot, Lyon.

## Electronic supplementary material

Additional file 1: **Results of the mixed-meal tolerance test: area under the curve C-peptide (nmol/l) of the different study groups stratified according to the duration of diabetes mellitus.** (DOC 34 KB)

Additional file 2: **Results of the mixed-meal tolerance test: peak C-peptide (nmol/l) of the different study groups stratified according to the duration of diabetes mellitus.** (DOC 34 KB)

## Abbreviations

AUCCP: Area under the curve C-peptide
GADAb: Glutamic acid decarboxylase autoantibody
IA-2Ab: Tyrosine phosphatase autoantibody
IQR: Interquartile range
LADA: Latent autoimmune diabetes in adults
MMTT: Mixed-meal tolerance test.
