# Supplementary material for: Insulin secretion in patients with latent autoimmune diabetes (LADA): half way between type 1 and type 2 diabetes: action LADA 9
Source: BMC Endocr Disord. 2015 Jan 9;15:1. doi: 10.1186/1472-6823-15-1 (PMC4297398; doi:10.1186/1472-6823-15-1)
Supplement: Supplementary file 1 — Additional file 1: Results of the mixed-meal tolerance test: area under the curve C-peptide (nmol/l) of the different study groups stratified according to the duration of diabetes mellitus. (DOC 34 KB) [file 12902_2014_301_MOESM1_ESM.doc]

**Additional file 1.** Results of the mixed-meal tolerance test: area under the curve C-peptide of the different study groups stratified according to the duration of diabetes mellitus

| Disease duration | Type 1 diabetes  (n = 33) | Type 2 diabetes  (n = 30) | LADA  (n = 32) | p^a^ | p^b^ |
| --- | --- | --- | --- | --- | --- |
| 6 – 18 m | 74.74 (63.93) | 292-25 (140.7) | 182.28 (106.73) | 0.023 | 0.218 |
|  | 57 (37.5 ; 103.4) | 242.7 (203.9 ; 373.3) | 194.7 (109.8 ; 281.1) |  |  |
| 18 m – 5 y | 32.48 (26.33) | 318.14 (116.82) | 136.22 (81.12) | 0.001 | 0.002 |
|  | 19.2 (19.2 ; 45.3) | 329 (249.9 ; 399.9) | 137.3 (74 ; 185) |  |  |
| 5 – 10 y | 20.48 (27.52) | 215.99 (87.27) | 109.92 (109.92) | 0.027 | 0.025 |
|  | 19.2 (3.6 ; 19.2) | 189 (160.6 ; 273.9) | 81.9 (16.6 ; 168.9) |  |  |

All results are given as mean (SD) and median (IQR). ^a^ p value for comparisons between type 1 diabetes and LADA; ^b^ p value for comparison between type 2 diabetes and LADA. The number of insulin treated type 2 diabetic subjects according to disease duration was 2, 3 and 3 in categories 6 –18 months, 19 months – 5 years, and 5 – 10 years, respectively. The corresponding distribution in patients with LADA was 5, 6 and 9, respectively.
