# Supplementary material for: Insulin secretion in patients with latent autoimmune diabetes (LADA): half way between type 1 and type 2 diabetes: action LADA 9
Source: BMC Endocr Disord. 2015 Jan 9;15:1. doi: 10.1186/1472-6823-15-1 (PMC4297398; doi:10.1186/1472-6823-15-1)
Supplement: Supplementary file 2 — Additional file 2: Results of the mixed-meal tolerance test: peak C-peptide (nmol/l) of the different study groups stratified according to the duration of diabetes mellitus. (DOC 34 KB) [file 12902_2014_301_MOESM2_ESM.doc]

**Additional file 2.** Results of the mixed-meal tolerance test: peak C-peptide (nmol/l) of the different study groups stratified according to the duration of diabetes mellitus

| Disease duration | Type 1 diabetes  (n = 33) | Type 2 diabetes  (n = 30) | LADA  (n = 32) | p^a^ | p^b^ |
| --- | --- | --- | --- | --- | --- |
| 6 – 18 m | 0,84 (0,63) | 3,26 (1,65) | 2,08 (1,14) | 0,011 | 0,165 |
|  | 0,7 (0,5 ; 1,2) | 2,5 (2,2 ; 3,7) | 2,2 (1,4 ; 3) |  |  |
| 18 m – 5 y | 0,34 (0,3) | 3,32 (1,2) | 1,44 (0,84) | 0,001 | 0,001 |
|  | 0,2 (0,2 ; 0,6) | 3,4 (2,5 ; 4,2) | 1,5 (0,8 ; 2) |  |  |
| 5 – 10 y | 0,21 (0,31) | 2,43 (0,83) | 1,06 (0,96) | 0,013 | 0,003 |
|  | 0,2 (0 ; 0,2) | 2,3 (2 ; 2,9) | 0,9 (0,2 ; 1,6) |  |  |

All results are given as mean (SD) and median (IQR). ^a^ p value for comparisons between type 1 diabetes and LADA; ^b^ p value for comparison between type 2 diabetes and LADA. The number of insulin treated type 2 diabetic subjects according to disease duration was 2, 3 and 3 in categories 6 –18 months, 19 months – 5 years, and 5 – 10 years, respectively. The corresponding distribution in patients with LADA was 5, 6 and 9, respectively.
